# Implementation of the Treat-to-Target Concept in Evaluation of Psoriatic Arthritis Patients

**DOI:** 10.3390/jcm10235659

**Published:** 2021-11-30

**Authors:** Tal Gazitt, Muhanad Abu Elhija, Amir Haddad, Idit Lavi, Muna Elias, Devy Zisman

**Affiliations:** 1 Rheumatology Unit, Carmel Medical Center, Haifa 3436212, Israel; Mahanedab@clalit.org.il (M.A.E.); haddadamir@yahoo.com (A.H.); munael@clalit.org.il (M.E.); devyzisman@gmail.com (D.Z.); 2 Division of Rheumatology, Department of Medicine, University of Washington Medical Center, Seattle, WA 98195, USA; 3 Department of Community Medicine and Epidemiology, Carmel Medical Center, Haifa 3436212, Israel; lavi_idit@clalit.org.il; 4 The Ruth and Bruce Rappaport Faculty of Medicine, Technion, Haifa 3525433, Israel

**Keywords:** psoriatic arthritis, assessment, disease activity, composite disease activity measures, patient-reported outcomes

## Abstract

Background: The treat-to-target approach was recently adopted for psoriatic arthritis (PsA) management. Objective: To assess the implementation of the “treat-to-target” (T2T) concept in daily management of PsA by use of composite scores of disease activity versus clinical judgement alone. Methods: A total of 117 PsA patients from a longitudinal PsA cohort were enrolled consecutively in the study during each patient’s first clinic visit during 2016–2017. Clinic notes from the treating rheumatologist were reviewed by an independent rheumatologist, noting clinical impression of disease activity, treatment changes based on clinical judgement, and rationale. Treatment changes were then compared to the use of formal disease activity parameters in Minimal Disease Activity (MDA) and Disease Activity Index for Psoriatic Arthritis (DAPSA) composite measures. All associations were assessed using the chi-square test or the Mann–Whitney test, as appropriate. Results: The 117 PsA patient cohort consisted of 65.5% women, mean age 58.4 ± 13.6 years. Clinical judgement of treating rheumatologist concorded with MDA and DAPSA in 76 (65.5%) and 74 (64.9%) patients, respectively. Agreement between clinical judgement and composite measure criteria did not correlate with patient age, sex, alcohol/tobacco use, or treatment regimens chosen. Disagreement between physician assessment and MDA occurred in 40 (34.5%) cases: in 30 cases, the MDA status was overestimated due to disregard of patient reported outcomes (PRO), while underestimation of MDA status occurred in 25% of cases with treatment changes made in patients with a single active joint or enthesis. Underestimation of disease activity using DAPSA occurred in 22 cases and could be attributed to disregarding tender joint count, patient pain visual analogue scale and C-reactive protein level. Conclusion: In our cohort, agreement between clinical impression and formal composite measure utilization for implementation of T2T strategy occurred in 65% of patients. Discordance resulted from physicians’ overlooking PRO and emphasizing objective findings when using clinical judgement alone.

## 1. Introduction

In many areas of medicine, it has been shown that following a predefined treatment goal, termed the treat-to-target (T2T) approach, is more helpful in reducing complications and organ damage than treatment based on clinical judgement alone [1,2]. This approach was adopted in the management of rheumatic diseases [3,4,5,6].

Psoriatic arthritis (PsA) belongs to seronegative spondyloarthropathies, a group of rheumatic diseases that have common genetic associations and share certain clinical features aside from peripheral arthritis, such as spondylitis, enthesitis, dactylitis, uveitis, and inflammatory bowel disease. PsA is associated with significant morbidity due to progressive joint damage, reducing patients’ health-related quality of life and functional capacity, compared to psoriasis patients or healthy controls [7,8]. Maintaining sustained minimal disease activity is of importance in PsA, as it is associated with low progression of radiologic joint damage over time [9].

The Group for Research and Assessment of Psoriasis and Psoriatic Arthritis (GRAPPA) published recommendations for PsA management with six overarching goals of therapy, including achievement of the lowest possible level of disease activity in all disease domains—arthritis, enthesitis, dactylitis, axial, skin, and nail involvement—in order to optimize functional status, prevent structural damage, and improve quality of life and well-being [10]. Given the multifaceted nature of PsA, it was noted that patients should be evaluated regularly and have treatment adjusted as needed in order to achieve these goals, with the current accepted main treatment target being remission or low disease activity to reduce inflammatory burden [11,12,13,14]. As no specific disease activity measure has been endorsed to date in the management of PsA, it is recommended to assess disease activity by using any one of the several disease activity measures addressing different domains of disease, including patient-reported outcomes (PRO) [11,12,13].

Currently, several valid composite measures exist for assessing disease activity in PsA [15], such as the PsA Disease Activity Score (PASDAS) [16,17], the Composite Disease Activity Index in PsA (CPDAI) [18], Minimal Disease Activity (MDA) [19,20,21], and the Disease Activity Index for Psoriatic Arthritis (DAPSA) score [22,23,24]. While these composite measures are being increasingly used in clinical research and observational studies [21,25,26,27,28], the relative concordance between their respective parameters and the parameters used by physicians in assessing disease activity in daily clinical practice is unknown.

The objective of our study was thus to assess the real-life implementation of the T2T concept in daily clinical practice using clinical impression vs. formal composite disease activity measure utilization, using MDA and DAPSA.

## 2. Methods

### 2.1. Study Population

PsA patients that fulfilled the Classificiation for Psoriatic Arthritis (CASPAR) criteria who were ≥18 years of age, who also agreed to participate in a longitudinal observational cohort study, were followed in a combined rheumatology–dermatology clinic at 6–12 month intervals, according to a standardized protocol that includes collection of clinical and laboratory data regarding patient demographics, self-reported formal disability status (i.e., receiving a living stipend from the Israeli National Social Security System due to formal recognition of disability from patient’s rheumatologic illness), clinical data with emphasis on skin, joints, entheses, and dactylitis involvement as well as PRO, laboratory data including markers of inflammation, and medication use. Each enrolled patient’s first clinic visit during 2016–2017 was included in this study. All patients were assessed by one of two rheumatologists (D.Z and A.H).

A third rheumatologist (M.A.H) was assigned to retrospectively review the clinic visit notes for all patient visits included in the cohort. Data extracted from each protocol visit note included patient demographics; alcohol and tobacco use; duration of PsA and psoriasis; and clinical manifestation, including 68 tender joint count (TJC), 66 swollen joint count (SJC), 16 enthesial and 20 dactylitis counts, skin involvement (Psoriasis Area and Severity Index, (PASI) or total body surface area (BSA)), patient PRO (patient visual analogue scale (VAS), patient global disease activity VAS (PtGA), and Health Assessment Questionnaire (HAQ)), C-reactive protein (CRP) levels, medication use, physician global assessment on a 0–10 numerical scale, and treatment changes and rationale recorded by the treating physician.

The evaluating rheumatologist (M.A.H) then calculated the MDA and DAPSA scores based on the data included in the clinic visit notes, reviewed treatment changes, and determined the concordance between clinical judgement and formal disease activity scores in assessing disease activity and need for treatment change.

MDA evaluated in this study is a valid composite disease activity measure representative of the multifaceted domains of psoriatic disease, including peripheral arthritis (tender and swollen joints) and enthesis and skin involvement. It also includes three categories of PRO: patient pain VAS, PtGA, and HAQ. MDA status is achieved when any five of the seven criteria are met, while patients are said to have very low disease activity, which could represent a state of remission, if all seven criteria are fulfilled [19]. Unlike MDA, which is a binary tool signifying active/inactive disease, the DAPSA, an additional valid composite disease activity measure used in this study, is a continuous measure of disease activity and has several cut-off values: remission (0–4), low (5–14), moderate (15–28), and high disease activity (>28). The DAPSA score includes peripheral arthritis involvement, CRP level, and two PRO categories (patient VAS and PtGA) [22,23,24].

In this study, proper implementation of T2T strategy for tight disease activity control was defined as the physician’s alteration of treatment regimen based on clinical judgement whenever the physician noted that patients were not in low disease activity or remission, or the physician’s recorded rationale for forgoing treatment alteration based on medication side effects, patient comorbidities, pregnancy, patient preferences, etc., in comparison with the formal use of MDA and DAPSA as validated composite measures as the target for disease management.

In our analysis, two cutoff values for the MDA score were used to analyze T2T adherence: MDA < 5 signifying active disease and MDA ≥ 5 signifying low disease activity or remission. For the purpose of T2T analysis using DAPSA, two different cutoff possibilities for the DAPSA score were evaluated—one in which remission and low disease activity levels were grouped together (DAPSA score ≤ 14) vs. moderate to high disease activity grouped together (DAPSA score > 14).

### 2.2. Statistical Analysis

Continuous data are presented as mean ± standard deviation (SD), while categorical variables are presented as numbers and percentages. The associations between proper T2T concept implementation and categorical and continuous variables were assessed by chi-square test or Mann–Whitney test, as appropriate. The association between physician’s assessment at each clinic visit with each of the MDA or DAPSA parameters was evaluated using chi-square test or Fisher’s exact test for small samples, as appropriate.

All data were analyzed using SPSS, version 24 (IBM SPSS Statistics for Windows, version 24.0, 2016, Armonk, NY, USA). All tests were two sided; *p* values of <0.05 were considered statistically significant.

All patients signed informed consent according to the declaration of Helsinki agreeing to participate in this PsA longitudinal cohort. The study was approved by the Institutional Review Board (IRB, also known as the Helsinki Committee) of Carmel Hospital (CMC 0044-11).

## 3. Results

### 3.1. Study Population Characteristics

A total of 117 consecutive patient visits of 117 different patients were evaluated; one patient visit was excluded from the analysis due to a lack of complete data in calculating MDA, and three patient visits were excluded from the analysis due to a lack of complete data in calculating DAPSA. The mean patient age was 58.4 ± 13.6 years, 74 (63.8%) of whom were women, with a mean age of 42.7 ± 13.0 years at PsA onset and 32.0 ± 16.3 years for psoriasis (Table 1). Most patients had at least one major comorbidity (84/116, 72.4%) chief among which was cardiovascular disease (63/116, 54.3%). A concurrent diagnosis of fibromyalgia syndrome (FMS) was present in 6/116 (5.2%) patients, and 16/116 (13.8%) had osteoarthritis (OA). Despite most patients having significant comorbidities, only 15/116 (12.9%) of patients self-reported formal recognition of disability status. Predominant PsA patterns were polyarthritis in 55/116 (47.0%), oligoarthritis in 36/116 (31.0%), axial involvement as a sole disease manifestation in 3/116 (2.6 %), dactylitis in 13/116 (11.2 %) and enthesitis in 64/116 (55.2%). The average PASI score was 2.0 ± 3.5. In none of the visits was a validated disease activity score used by the treating physician, and treatment changes were based on the physician’s clinical impression of disease activity.

### 3.2. T2T Implementation Using MDA and DAPSA Scores versus Clinical Judgement

After reviewing patient visit notes, the independent assessing rheumatologist concluded that agreement between implementation of T2T strategy using clinical judgement versus using MDA criteria occurred only in 76/116 (65.5%) cases, and was not affected by patient age, sex, alcohol or tobacco use, as well as the various treatment regimens ((conventional synthetic disease modifying anti-rheumatic drugs (csDMARD) versus biologic DMARD (bDMARD)) (Table 1). Physician assessment of disease activity did not correlate with the MDA score in assessment of 40 (34.5%) patients (Table 2). In 30/40 (75.0%) of cases, the patients’ MDA status was overestimated, so patients were considered in MDA due to disregard of the PRO categories of the MDA score, including 29 patients reporting a high VAS pain score, 22 patients reporting a high PtGA, and 25 patients reporting a high HAQ score (Table 3). Conversely, patient achievement of MDA status was underestimated in 10/40 (25.0%) of cases in which treatment changes were made by the treating physician based on a single involved joint/enthesis, in discordance with the MDA composite measure criteria in which inflammation in a single joint/enthesis is still considered low disease activity/remission (Table 3). Similarly, the independent assessing rheumatologist concluded that concordance between implementation of T2T strategy using clinical judgement versus using DAPSA criteria occurred in 74/114 (64.9%) of patients (Table 2), with underestimation of disease activity on the part of the treating physician occurring in 22/40 (55.0%) patients due to the overlooking of subjective findings (tender joint count, and patient VAS) as well as the CRP level (Table 3). Overestimation of disease activity occurred in 18/40 cases with no specific component of the DAPSA composite measure being overlooked in this type of inaccurate physician impression in a statistically significant manner (Table 3).

## 4. Discussion

In our study, we found that there is limited agreement between the formal use of composite disease activity measures and physician clinical impression of disease activity in the management of PsA. Tight T2T control using clinical judgement alone was implemented in actuality in about 65.0% of PsA patients when compared to using a validated disease activity measure (MDA or DAPSA).

In searching the literature, we found only a single study by van Mens et al. [29] that compared the use of MDA to clinical judgement in assessing disease activity in PsA in daily life. As in our study, the study by van Mens et al. incorporated an independent rheumatologist to evaluate whether the T2T approach was being implemented by providers, and was able to show that only about 35.0% (88/250) of PsA patients considered by the treating rheumatologist to have “acceptable disease state” actually fulfilled MDA status. Similar to results from our study, factors contributing to this discrepancy were the under-estimation of the “subjective” components of the composite measures, such as tender joint count and patient pain and global disease activity scores. Additionally, our study also showed that the tendency to overemphasize “objective” clinical findings, such as the involvement of a single joint or enthesis, by treating physicians relative to PRO categories led to the underappreciation of MDA status when it was met in actuality. Similarly, we previously demonstrated this overemphasis on “objective” measures of disease activity in our study on T2T adherence in RA management [30]. 

The heterogeneity of PsA and lack of consensus on which validated disease activity measure to use have hampered agreement on the most appropriate target to use in the T2T strategy in PsA, ref. [31] leaving clinicians to individually choose which disease activity measure/s to follow in their attempt at T2T implementation in daily practice. As we and van Mens et al. were able to demonstrate in our respective studies, the lack of use of a pre-specified, simple and validated disease activity measure leaves physicians in daily clinical practice in the position of relying on “objective” disease activity measures which can be quantitatively measured, such as swollen joints or entheses, while overlooking “subjective” patient-reported components of disease activity. We surmise that this tendency to rely on “objective” disease activity measures that can be quantitatively measured while underestimating the significance of PRO categories in assessing disease activity likely stems from the desire by the treating physicians to avoid making changes in treatment regimens based on “subjective” disease activity measures, which may be distorted by the presence of co-existing conditions, such as osteoarthritis or fibromyalgia syndrome (FMS), noted in the literature to have high prevalence in PsA [32,33] and to affect PRO and composite disease activity scores, including MDA and DAPSA [33]. Moreover, compounding the difficulty in assessing disease activity in PsA is the lack of concordance between the clinician and PsA patient perspectives on the definition of low disease activity or remission, as recently shown by Gorlier et al. [34].

Barriers to proper T2T implementation in daily clinical practice may also stem from lack of time for complete clinical evaluation of multiple disease domains in PsA and lack of existence of a single, simple, universally accepted and reliable disease activity score capturing all disease domains of PsA. Indeed, a recent review on challenges in measuring PsA disease activity highlights the difficulty in evaluating 68 joints in PsA rather than 28 joints required by the Clinical Disease Activity Index (CDAI) score used in rheumatoid arthritis (RA) and the need to measure multiple domains for disease activity in PsA [35]. The issue of time constraints was recently highlighted in an online survey of 439 U.S. rheumatologists discussing barriers to implementation of T2T strategy in clinical practice, noting time constraints in daily practice (62.5%) and a sense of inefficiency of having to report metrics in electronic medical records (34.8%) [36]. This issue has even led to the recent suggestion to identify a ‘target-to-treat’ of a specific aspect or few aspects of disease most significant to each individual PsA patient as an alternative appropriate strategy rather than attempting to cover all disease domains [31].

Given the significant improvements in both PsA disease activity and patient-related outcomes in utilizing a tight T2T approach in the management of PsA as demonstrated by the TICOPA trial [27], there is significance in reaching a consensus on which validated disease activity measure to use in the management of PsA in daily practice.

Limitations of our study include the small number of assessing physicians from only one medical center. In addition, we did not capture axial involvement due to the focus on MDA and DAPSA composite measures, which lack assessment of axial involvement, although axial involvement may have prompted treatment changes. The strengths of our study lie in the relatively large number of consecutive PsA patient visits included in our analysis of real-life implementation of T2T strategy in PsA as evaluated against the use of two different practical composite measures.

## 5. Conclusions

In our cohort, the T2T concept, using a validated score as the target, was implemented properly in approximately 65.0% of PsA patients due to reliance on physicians’ clinical impression of disease activity. The main obstacle we encountered in implementation of the T2T concept was in physicians overlooking the PRO components and over-emphasizing the “objective” components of the scores when using clinical judgement alone. In order to improve treatment outcomes in daily practice, efforts are needed to increase physician awareness regarding the significance of PRO categories of disease activity and the use of validated scores in assessing disease activity.

### Key Points

1. The T2T concept is properly implemented in the management of about 65.0% of PsA patients when compared to the use of formal composite disease activity measures in daily clinical practice.
2. Discordance between clinical impression and actual disease activity level lies in physician reliance on “objective” components of disease activity, such as swollen joints and entheses, and disregard of more “subjective” aspects of disease activity assessment, such as PRO.
3. There is an unmet need for having a pre-specified, simple, practical, and valid disease activity score which may be used in the management of PsA patients in daily clinical practice.

## Figures and Tables

**Table 1:** Study population characteristics.

Parameter		T2T	T2T Not	Total	*p* Value
		Implemented *	Implemented *		
		N = 76	N = 40	N = 116	
**Age** Mean (±SD)	Age at baseline	57.7 ± 12.5	59.9 ± 15.5	58.4 ± 13.6	NS
	Age at onset of PsO	31.0 ± 15.2	33.6 ± 18.4	32.0 ± 16.3	NS
	Age at diagnosis of PsO	34.0 ± 15.4	38.3 ± 17.3	35.5 ± 16.1	NS
	Age at PsA onset	42.2 ± 12.1	43.9 ± 14.2	42.7 ± 13.0	NS
	Age at diagnosis PsA	45.2 ± 12.1	47.1 ± 13.6	45.8 ± 12.8	NS
**Sex**	Female	47	27	74	NS
		61.80%	67.50%	63.80%	
**Ethnicity**	Jewish	70	36	106	NS
		92.10%	90%	91.40%	
	Arabs	4	4	8	NS
		5.30%	10.00%	6.90%	
**Smoking**	Ever	18	6	24	NS
		23.70%	15.00%	20.70%	
**Alcohol use**	Ever	27	10	37	NS
		35.50%	25.00%	31.90%	
**Comorbidities**	Overall	56	28	84	NS
		73.70%	70.00%	72.40%	
	Cardiovascular	44	19	63	NS
		57.90%	47.50%	54.30%	
	Hypertension	13	7	20	NS
		17.10%	17.50%	17.20%	
	Diabetes mellitus	13	11	24	NS
		17.10%	27.50%	20.70%	
	Hyperlipidemia	34	13	47	NS
		44.70%	30.20%	40.50%	
	Osteoarthritis	10	6	16	NS
		13.20%	15.00%	13.80%	
	Fibromyalgia	4	2	6	NS
		5.30%	5.00%	5.20%	
**Clinical parameters** No. patients, % (Mean ± SD)	Tender joints	42, 55.3%	22, 55.0%	64, 55.2%	NS
		(4.3 ± 6.9)	(2.9 ± 4.7)	(3.8 ± 6.6)	
	Swollen joints	44, 57.9%	30, 75.0%	74, 63.8%	NS
		(2.9 ± 4.7)	(1.4 ± 2.7)	(2.4 ± 4.2)	
	Dactylitis	9, 11.8%	4, 10.0%	13, 11.2%	NS
		(0.2 ± 0.6)	(0.1 ± 0.4)	(0.2 ± 0.5)	
	Enthesitis	40, 52.6%	24, 60%	64, 55.2%	NS
		(3.5 ± 5.4)	(3.1 ± 5.4)	(3.4 ± 5.4)	
	PASI	35, 46.1%	28, 70.0%	63, 54.3%	NS
		(2.4 ± 4.0)	(1.2 ± 2.1)	(2.0 ± 3.5)	
**Assessment** **questionnaires**	Patient pain VAS	21, 27.6%	11, 27.5%	32, 27.6%	NS
		(4.5, ± 3.1)	(4.4 ± 3.4)	(4.5 ± 3.2)	
	PtGA	25, 32.9%	13, 32.5%	38, 32.8%	NS
		(4.2 ± 2.7)	( 4.5 ± 3.3)	(4.3 ± 3.1)	
	HAQ	30, 39.5%	17, 42.5%	47, 40.5%	NS
		(0.9 ± 0.8)	(0.8 ± 0.7)	(0.9 ± 0.7)	
**Medications**	Methotrexate	29	15	44	NS
		38.20%	37.50%	37.90%	
	Cyclosporine	1	1	2	NS
		1.30%	2.50%	1.70%	
	Sulfasalazine	5	0	5	NS
		6.60%	0.00%	4.30%	
	Hydroxychloroquine	0	0	0	-
		0.00%	0.00%	0.00%	
	Leflunomide	4	2	6	NS
		5.30%	5.00%	5.20%	
	Apremilast	10	3	13	NS
		13.20%	7.50%	11.20%	
	Golimumab	7	2	9	NS
		9.20%	5.00%	7.80%	
	Infliximab	3	1	4	NS
		3.90%	2.50%	3.40%	
	Adalimumab	10	8	18	NS
		13.20%	20.00%	15.50%	
	Etanercept	15	11	26	NS
		19.70%	27.50%	22.40%	
	Ustekinumab	5	3	8	NS
		6.60%	7.50%	6.90%	
	Secukinumab	10	7	17	NS
		13.20%	17.50%	14.70%	
	Corticosteroids	5	1	6	NS
		6.60%	2.50%	5.20%	
	cDMARDs	38	17	55	NS
		50.00%	42.50%	47.40%	
	bDMARDs	52	33	85	NS
		68.40%	82.50%	73.30%	

Abbreviations: b/c DMARDs = biologic/conventional disease-modifying anti-rheumatic drugs, HAQ = Health Assessment Questionnaire, N = number of patients, NS = not significant, PASI = Psoriasis Area and Severity Index, PsA = psoriatic arthritis, PsO = psoriasis, PtGA = Patient Global Assessment of Disease Activity, SD = standard deviation, T2T = Treat to Target, VAS = Visual Analogue Scale. * Treat to Target (T2T) implemented or not implemented based on comparison of clinical judgement to validated minimal disease activity (MDA) score.

**Table 2:** The degree of concordance between treatment decision. Based on clinical judgment to validated MDA and DAPSA scores.

	Physician Impression and Decision				
	No Active Disease No Treatment Changes	Active Disease Treatment Changed	Active Disease No Treatment Changes	Active Disease No Treatment Changes Due to Physician/Patient Decision (Noted in Chart)	Total Number of Patients
**MDA < 5**	30 56.6% Incorrect clinical decision (Underestimation)	40 83.3% Correct clinical decision	2 100.0% Incorrect clinical decision (Overestimation)	11 83.3% Correct clinical decision	83 71.3%
**MDA** **≥ 5 (Remission)**	23 43.4% Correct clinical decision	8 16.7% Incorrect clinical decision (Overestimation)	0 0.0% Correct clinical decision	2 16.7% Correct clinical decision	33 28.7%
**Total**	53 100.0%	48 100.0%	2 100.0%	13 100.0%	116 100.0%
**DAPSA > 14 (Active disease)**	21 40.4% Incorrect clinical decision (Underestimation)	30 62.5% Correct clinical decision	1 50.0% Incorrect clinical decision (Underestimation)	10 83.3% Correct clinical decision	62 54.4%
**DAPSA** **≤ 14 (Low disease activity to Remission)**	31 59.6% Correct clinical decision	18 37.5% Incorrect clinical decision (Overestimation)	1 50.0% Correct clinical decision	2 16.7% Correct clinical decision	52 45.6%
**Total**	52 100.0%	48 100.0%	2 100.0%	12 100.0%	114 100.0%

T2T implemented—blue;T2T not implemented—red; Abbreviations: MDA = Minimal Disease Activity; Abbreviations: DAPSA = Disease Activity Index for Psoriatic Arthritis.

**Table 3:** Factors influencing discordance between physician clinical impression and individual MDA and DAPSA score components.

	Underestimation * MDA < 5 30 Patients (%)	Overestimation # MDA ≥ 5 10 Patients (%)	*p*-Value	Overestimation # DAPSA ≤ 14 18 Patients (%)	Underestimation * DAPSA > 14 21 + 1 Patients (%)	*p*-Value
TJC	16 (53.3%)	2 (20.0%)	NS	0.6 ± 0.9	4.0 ± 7.4	0.03
SJC	8 (26.7%)	2 (20.0%)	NS	0.8 ± 1.0	1.5 ± 2.8	NS
PASI	8 (20.7%)	4 (40.0%)	NS	N/A	N/A	N/A
Tender entheseal points	15 (50.0%)	1 (10.0%)	0.03	N/A	N/A	N/A
Patient pain VAS	29 (96.7%)	0 (0.0%)	<0.0001	1.9 ± 32.0	7.1 ± 2.0	<0.0001
PtGA, n (%)	25 (83.3%)	2 (20.0%)	0.001	3 (16.7%)	7 (31.8%)	NS
HAQ	22 (73.3%)	1 (10.0%)	0.001	N/A	N/A	N/A
CRP	N/A	N/A	N/A	2.2 ± 2.7	5.2 ± 5.6	0.02

Abbreviations: CRP = C-reactive protein, DAPSA = Disease Activity Index for Psoriatic Arthritis, HAQ = Health Assessment Questionnaire, MDA = Minimal Disease Activity, N/A = not applicable, NS = non-significant, PASI = Psoriasis Area and Severity Index, PtGA = patient global assessment, SJC = Swollen Joint Count, TJC = Tender Joint Count, VAS = Visual Analogue Scale. For MDA assessment (a dichotomous, binary measurement), the following were taken into consideration: TJC > 1, SJC > 1, PASI > 1, Tender entheseal points > 1, Patient Pain VAS > 1.5, PtGA > 2, HAQ > 0.5. For DAPSA assessment (a continuous measurement), the following were taken into consideration: TJC, SJC, CRP, PtGA > 2, Patient pain VAS. * Underestimation = physician accidentally thought disease activity was lower than it really was; # Overestimation = physician accidentally thought disease activity was higher than it really was.

## Data Availability

All data are available upon request.
